# Automated Tracking of Animal Posture and Movement during Exploration and Sensory Orientation Behaviors

**DOI:** 10.1371/journal.pone.0041642

**Published:** 2012-08-09

**Authors:** Alex Gomez-Marin, Nicolas Partoune, Greg J. Stephens, Matthieu Louis

**Affiliations:** 1 European Molecular Biology Laboratory/Center for Genomic Regulation Systems Biology Unit, Center for Genomic Regulation & Universitat Pompeu Fabra, Barcelona, Spain; 2 Department of Electrical Engineering and Computer Science, Université de Liège, Liege Sart-Tilman, Belgium; 3 Joseph Henry Laboratories of Physics & Lewis-Sigler Institute for Integrative Genomics Princeton University, Princeton, New Jersey, United States of America; Imperial College London, United Kingdom

## Abstract

**Background:**

The nervous functions of an organism are primarily reflected in the behavior it is capable of. Measuring behavior quantitatively, at high-resolution and in an automated fashion provides valuable information about the underlying neural circuit computation. Accordingly, computer-vision applications for animal tracking are becoming a key complementary toolkit to genetic, molecular and electrophysiological characterization in systems neuroscience.

**Methodology/Principal Findings:**

We present Sensory Orientation Software (*SOS*) to measure behavior and infer sensory experience correlates. *SOS* is a simple and versatile system to track body posture and motion of single animals in two-dimensional environments. In the presence of a sensory landscape, tracking the trajectory of the animal's sensors and its postural evolution provides a quantitative framework to study sensorimotor integration. To illustrate the utility of *SOS*, we examine the orientation behavior of fruit fly larvae in response to odor, temperature and light gradients. We show that *SOS* is suitable to carry out high-resolution behavioral tracking for a wide range of organisms including flatworms, fishes and mice.

**Conclusions/Significance:**

Our work contributes to the growing repertoire of behavioral analysis tools for collecting rich and fine-grained data to draw and test hypothesis about the functioning of the nervous system. By providing open-access to our code and documenting the software design, we aim to encourage the adaptation of *SOS* by a wide community of non-specialists to their particular model organism and questions of interest.

## Introduction

In a similar way that detailed knowledge of the dynamics of ion channels enhance our understanding of neurons, precise behavioral characterizations help to unravel the function of neural circuits. However, natural behaviors are usually complex, variable and multidimensional, with no universal language such as that of action potentials. Therefore, quantifying behavior at high-resolution, efficiently, and in an unbiased fashion remains a challenge in most neurobiological studies [1]. Indeed, though manual annotation is common, *ad hoc* performance indices defined by the experimenter may fail to capture the information relevant to the transformation of sensory input into behavioral output. An alternative approach consists in measuring unconstrained behavior from its most fundamental components — the time course of the animal's posture — to search for principles simplifying the apparent complexity of the phenomenon [2]. This requires new techniques to systematically collect and analyze behavioral data.

Computer-vision offers a fundamental tool in the study of animal behavior. Several companies provide commercial software specifically devised to study a particular paradigm (e.g. the Morris water maze for rodents). Although these solutions can be onerous and difficult to customize, they have the advantage of working out of the box for the specific tasks they were designed for. In addition, a series of custom-made tracking software written by neurobiologists is now available, enabling behavioral measurements of individual animals at an unprecedented resolution in nematodes [3], [4], [5], flies [6], [7], [8], [9] and rodents [10]. Software capable of tracking multiple animals simultaneously [11], [12], [13] has augmented the toolkit for high-throughput screening. While the use of these tools is becoming common practice, it takes considerable effort to adapt and extend the codes to different behavioral paradigms or model organisms. We believe that there exists a scope for free, simple, and customizable software between sophisticated freeware and commercial packages.

Multipurpose tracking systems that measure motor responses and simultaneously infer the corresponding sensory input during unconstrained orientation behavior are scarce. To assess the sensory information accessible to an animal, it is important to determine not only the position of the center of mass (the animal being described as a moving dot in space), but also its posture and the kinematics of specific points along the body. For instance, while olfactory inputs are collected by sensors at the tip of the head in *Drosophila* larva, thermosensory and visual inputs arise from sensory neurons covering the whole body [14], [15], [16]. Similarly, escape responses and turning maneuvers in fishes involve intricate muscle activity patterns where body curvature and tail acceleration play a key role. In general, it is valuable to know not only where the animal is located in space but also what inputs are stimulating its sensors (visual and otherwise) together with its relative orientation to particular landmarks or other organisms.

Here we have developed Sensory Orientation Software (*SOS*) to extract and analyze fine-grained information about the posture and motion of single animals behaving in sensory landscapes. The *SOS* system consists of a series of custom-made Matlab codes for online animal tracking and offline processing and analysis. We provide access to all our scripts as File S1. The scripts are commented and documented in a step-by-step tutorial. We provide a test dataset (File S1) and include a movie illustrating the application of *SOS* to track different animals (Movie S1). Our software targets a community of non-experts in computer-vision or programming: it offers a flexible basis adaptable to several paradigms and organisms. Together with an accompanying manuscript by Colomb *et al.*, this work presents a free, customizable and pedagogical tool for behavioral tracking and analysis.

The structure of the paper is as follows. First, we describe the online tracking system. We estimate relevant spatial, temporal and data constraints related both to the animal's characteristics and the tracking procedure itself. Next, we explain how to compute postures from raw body shape images. We illustrate this approach in fruit fly larvae, flatworms, fish and mice. In the *Drosophila* larva, we show how to accurately infer the sensory stimuli to which particular loci along the larval body are exposed in a sensory landscape. We validate our approach by examining, at high-resolution, the sensorimotor trajectories of larvae in odor, temperature and light gradients.

## Materials and Methods

### Fly stocks and animal preparation

Fly stocks were maintained on conventional cornmeal-agar molasses medium at 22°C and kept in a 12 h dark-light cycle. The *Drosophila melanogaster* Canton-S strain was used as ‘wild type’. In all behavioral experiments, 6-day-old third instar foraging larvae were tested during the day. Room temperature was kept between 21 and 23°C and relative humidity between 50 and 60%. Larvae were washed from food medium by pouring a solution of 15% sucrose in the food vial. Individuals floating at the surface of the sucrose solution were transferred to the arena for behavioral tracking. Single animals were monitored while crawling on a 3% agarose slab.

### Sensory landscapes

Orientation behavior in *Drosophila* larvae was tested in controlled odor, temperature and light gradients. For chemotaxis, an airborne odor gradient was created by loading an odor droplet on the condensation ring of the lid of a 96-well plate, which was inverted on a surface of agarose to form a closed arena [17]. Ethyl butyrate (CAS number 105-54-4) was used as odorant. The odor concentration was estimated via infrared spectrometry [17]. For thermotaxis, a linear spatial thermal gradient was created with two thermoelectric temperature controllers (TC-48-20, TE Technology) maintaining the two extremes of the plate at different constant temperatures. The agarose layer was placed right on top of the metal plate and its temperature was directly measured with a thermometer (MM2000 Handhold Thermometer, TM Electronics). The experiment started after the establishment of a stationary gradient in the agar. For phototaxis, a bright light pad (5000 Kelvin color temperature radiation, Slimlite Lightbox, Kaiser) was placed perpendicularly to the agarose layer surface where the animal crawled, creating a sideways gradient.

### Behavioral arenas

A video camera (Stingray Camera, Allied Vision Technologies; Computar lens, 12–36 mm, 1∶2∶8, 2/3″ C) fixed on a stand was used to monitor larval behavior. Larval tracking lasted a maximum of five minutes and was interrupted when the animal left the field of view. Frames were streamed at 7 Hz live from the camera by the Image Acquisition toolbox of Matlab (The MathWorks, Natick, USA), which automatically recognizes DCAM compatible FireWire cameras upon installation of the CMU 1394 Digital Camera Driver. The installation of the Image Processing toolbox of Matlab is necessary to ensure the functionality of *SOS*. To maximize the effectiveness of the image processing, different conditions of illumination were designed to study each modality. For chemotaxis, a light pad (5000 Kelvin color temperature radiation, Slimlite Lightbox, Kaiser) illuminated the arena from above creating uniform daylight conditions, while the camera recorded images from below. This configuration allowed us to reduce the shadow from the condensation rings of the lid of the arena. For thermotaxis, the camera was placed above the agarose layer and the setup was illuminated by sideways red LEDs (620 nm wavelength, 30 lm luminous flux, Lumitronix LED, Technik GmbH). For phototaxis, the camera was placed above the agarose layer and sideways white-light illumination (Slimlite Lightbox, Kaiser) was sufficient to enable tracking. The light intensity was assumed to decay from the source position.

## Results

### Online tracking: from video streaming to animal postures

**Figure 1:**
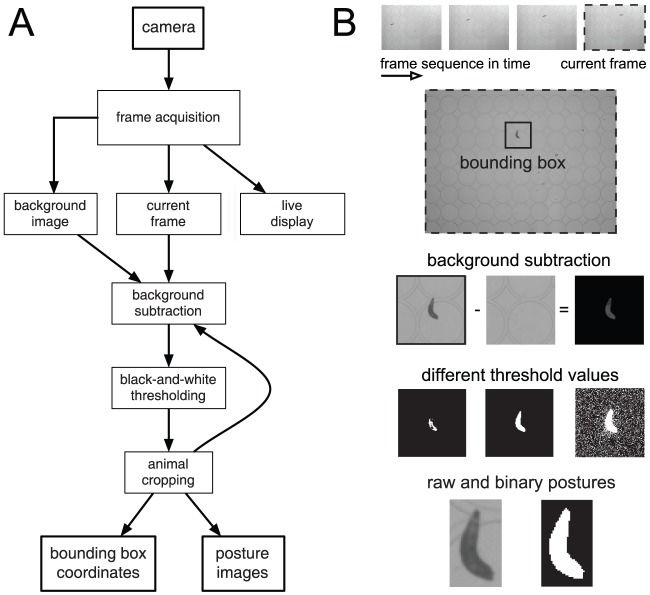
From experiments to animal shapes. (**A**) Flowchart of the sequential steps of the online software. (**B**) Illustration of the image processing. The quality of the object segmentation depends critically on the threshold used to binarize the image. Frames streamed from the camera are used to reconstruct the background, detect the animal, and track its motion. The body posture and location of points of interest are saved as the animal freely moves in the arena.

During online tracking camera frames are acquired live and preprocessed. Raw images of the animal in the arena are cropped into a bounding box enclosing the two-dimensional projection of its body shape. Together with the coordinates of the box, the sequence of cropped images is saved for offline analysis, optimizing the storage of raw data and making the subsequent processing more efficient. The operations allowing monitoring the behavior of a single animal in real time are schematized in the flowchart diagram of Figure 1.

The program is initiated by typing *track(x,y)* in the command prompt, where the argument *x* specifies the sampling interval in seconds and *y* is the total number of frames to be tracked. The tracking frequency is controlled by a built-in timer function. Messages are displayed at the prompt guiding the user and indicating the status of the tracker. The software connects to the camera and displays a live image of the arena on screen so that the experimenter can adjust, amongst other features, the field of view, the focus and the illumination intensity. This is important since the target recognition procedure is based on differences in contrast. The tracker detects the animal as the largest object with the highest contrast (darkest or brightest, depending on the illumination and the arena). The input grayscale frame is converted into a binary black-and-white image based on a threshold operation, whose value can be iteratively and automatically adjusted by the user before tracking starts.

Before capturing the body contour to be used for posture tracking, a steady background image of the whole field of view is acquired. By subtracting the background from the current image, the animal posture can be automatically and robustly segmented. Depending on the illumination, the stimulus delivery system, and the particularities of the arena, segmentation can be either straightforward or more complex. In the simplest case, the animal is the only salient object and the background is essentially uniform. Then, no background subtraction is necessary. When static objects are present in the image, the background can be built as an average over the whole time sequence [18]. However, that solution only works for offline tracking. Provided that there are no slight displacements of the arena during the experiment, saving an initial snapshot of the background before the animal is introduced in the arena should work. More generally, *SOS* reconstructs the background after the animal is loaded in the arena via the following procedure: it detects the animal as the largest salient object; it crops a small box around it; it saves the outer image; finally, it completes the outer image with the inner image of the bounding box once the animal has moved away from it. This prevents confounding the animal with water droplets, small pieces of dirt, and shades produced by the arena. Animal postures can be faithfully tracked anywhere in the arena (see Text S1).

The background is subtracted from the current frame and the remaining grayscale image is transformed through a threshold into a binary (black and white) image. Unsuitable thresholds can lead to fragmented or noisy binary postures. Even with the appropriate threshold, the binary image may still contain undesirable objects due to pixel noise artifacts or impurities in the substrate. Since these objects are usually smaller than the animal, they are easily erased by retaining only the largest object in the image via standard Matlab functions. When an image of the animal shape is the only object left, a smooth contour is easily obtained offline.

Once the tracker has successfully detected the animal from the background, it creates a small bounding box around it. This region of the arena corresponds to the most likely area where the animal will be found in the next frame. From then on, computations will only take place in that region of interest, speeding up the image processing. The software enters a loop where the animal shape is found and saved, together with the coordinate positions of the cropped bounding box in the arena system of reference. Tracking stops when the animal leaves the field of view, or if the preset sampling frequency is faster than the time the computer takes to acquire and process each frame. Data acquisition can also be terminated at any time by following the prompts generated by the program. At the end of each trial, the data (background, postures and coordinates) are saved in a folder named after the current date and time of the experiment.

### Getting to scale: selecting appropriate temporal and spatial resolutions of a tracking experiment

**Figure 2:**
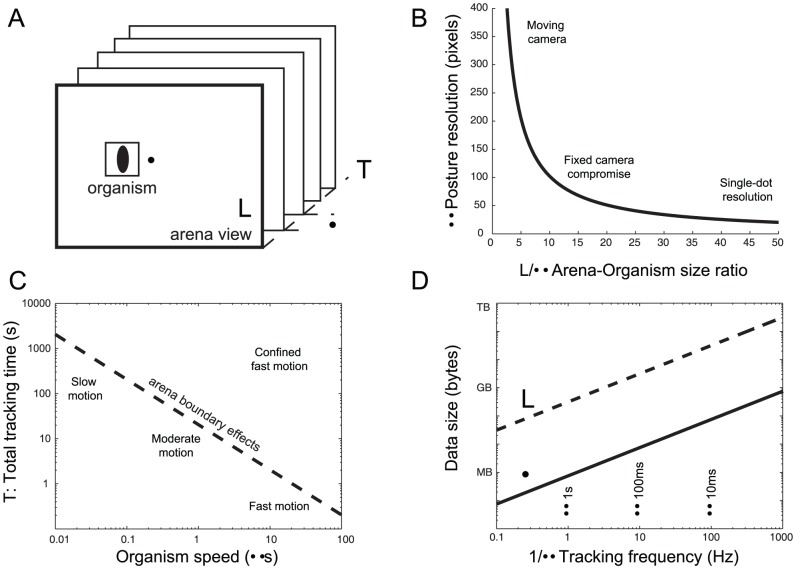
Spatial and temporal scales of a tracking experiment. (**A**) Scheme of the main spatial and temporal scales of the tracking: organism typical size (λ), behavioral arena field of view length (L), temporal resolution of the tracking (τ), and total time of the experiment (T). (**B**) Scaling of the organism posture resolution in pixels as a function of the relative field of view (considering a 1024-pixel frame resolution in length). Labeled areas represent different resolution limits. (**C**) Tracking time before the animal reaches the arena's edge (corresponding to an arena/organism ratio of 20 at a posture resolution of 50 pixels) as a function of the typical motion speed of an organism expressed in body length units. Labeled areas represent different locomotion speeds in relationship to the time to reach the boundaries of the arena. (**D**) Total data storage requirements for experiment lasting 5 minutes in the conditions of the previous panel, as a function of the tracking frequency in frames per second. The continuous line corresponds to disk space usage to save only a bounding box enclosing the organism posture, while the dashed line involves saving the complete frame, which considerable increases the storage needs.

Spatial resolution, temporal resolution and data storage requirements are mutually dependent. It is therefore convenient to estimate the tracking resolution limits given the arena constraints, animal features, and hardware-software computational characteristics (Figure 2A). These typical scales can be estimated, related and exploited in a useful manner. We summarize the most relevant relationships in Figure 2 as a guide for behavioral tracking experiments.

The level of detail at which the animal posture is tracked determines the number of body pixels to be processed and saved, therefore setting a trade-off between posture resolution, tracking frequency, and data size. Along the same lines, zooming in to acquire higher resolution postures implies tracking a smaller field of view, thus restricting the area where the animal can be tracked (Figure 2B). The animal-to-arena ratio provides a useful quantity to be related to the typical speed of the animal, image size, and frames per second. How fast the animal moves imposes a lower bound on the tracking speed and an upper bound on the total tracking time before the animal is likely to contact the arena boundaries (Figure 2C). In turn, given a particular posture resolution, a field of view and tracking frequency, the duration of the experiment will determine the total amount of data to be saved for that particular experiment (Figure 2D).

Spatial constraints related to animal posture resolution and motion in the arena arise mainly for setups where the camera is fixed. In closed-loop tracking systems where the camera follows the animal, very high postural resolution can be achieved in large arenas [19], [20]. Spatial and temporal constraints of the online tracking can be relaxed by acquiring frames as fast as needed without any preprocessing, at the expense of generating large volumes of video data. Sequences of high-resolution raw images for a single experiment can easily fill gigabytes of disk space. Our system is well suited to minimize the data stored upon completion of an experiment, saving only the relevant information as the experiment takes place, reducing the volume of data storage and making the subsequent offline analysis more efficient.

### Offline processing: analyzing sequences of animal posture

**Figure 3:**
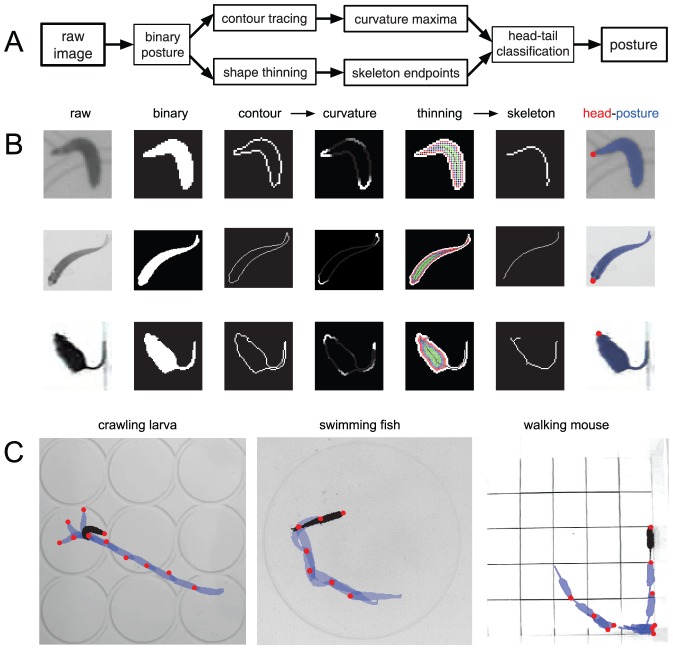
Postures in motion. (**A**) Flow chart of the sequential image processing steps to obtain high-resolution trajectories of animal postures. (**B**) Visual representation of the step schematics in (A) for three different organisms: fruit fly larvae, fish and mice. One can either perform a thinning operation on the binary image of the animal shape to find a skeleton whose endpoints will correspond to head and tail, or alternatively compute contour curvature maxima to determine the position of the head and tail. (**C**) Illustration of the tracking method for a temporal sequence of postures (blue silhouettes) and head positions (red dots) for a crawling larva, a swimming fish and a walking mouse.

During the offline analysis, each image is automatically processed to obtain animal-centric posture descriptors and loci of interest. The main steps to obtain postures are depicted in Figure 3A. We show how similar principles and operations can be applied for the high-resolution tracking of animals as distinct as fruit fly larvae, fishes or mice.

From the raw frames collected during the experimental session, the goal now is to generate high-resolution trajectories of the animal's posture that quantitatively describe the time course of its behavior. In short, we automatically trace the animal contour to compute its curvature and skeleton, in order to extract the position of several points of interest, including head and tail.

Once the original image has been segmented and binarized, the body edge is detected as the boundary between black and white pixels. If necessary, the shape can be smoothed by standard image processing procedures (see Text S1). Next, we find the skeleton using a thinning operation, which recursively removes pixels from the boundary until a single-pixel-wide connected skeleton is obtained (Figure 3B). The body skeleton is convenient for two reasons. First, it gives access to the local curvature of the animal along its anterior-posterior axis — a representation which permitted to make fundamental discoveries in *C. elegans* [2]. Second, the two end-points of the skeleton can be used as a proxy for animal head and tail positions, as is discussed below. The thinning process can produce spurious branches that lead to skeletons with more than two endpoints. The code keeps track of the fraction of such cases. If the contour is smooth and regular, as is the case for the *Drosophila* larva, spurs are rare and problematic frames can be discarded from the analysis. Depending on the particular organism that is being tracked, one could make use of such extra branches of the skeleton to detect relevant posture features such as legs, wings or fins. Alternatively, a second approach to obtain head and tail positions relies on the identification of points of maximum curvature along the animal's contour (see Figure 3B). The skeleton is then built from such points by tracing bisectors along left and right sides of the animal perimeter [13].

Differentiating the head from the tail is necessary to robustly reconstruct trajectories. When the temporal resolution is high, classification is achieved via a simple “distance rule”: the head in the current frame is identified as the closest locus to the head in the previous frame. This rule only requires human intervention to define the head position in the first frame. The program displays the first grayscale image of the animal on the screen and asks the user to click first near the head and then near the tail positions. From then on, head and tail are sorted automatically. This simple clustering algorithm can robustly detect the head position even if the animal is not moving or if it is engaged in backward locomotion. The algorithm only requires a tracking frequency that is faster than the typical speed of motion expressed in units of body length (Figure 2). When the animal bends to such an extent that a blob-like shape is created, the head and the tail can be swapped. We automatically flag these events during the first round of processing to allow for a correction if necessary (see Text S1). Furthermore, a visual animation is displayed at the end of the image processing so that the user can easily review any potential problems in loci assignment. For all frames associated with a potential error, the program pauses and invites the user to disambiguate the classification.

To illustrate the use of *SOS*, we tracked the postural dynamics of four model organisms in neurobiology: a fruit fly larva (third instar *Drosophila melanogaster* larva) crawling on an agarose surface, a flatworm (planarian *Schmidtea mediterranea*) swimming away from a light source, a fish (adult zebra fish *Danio Rerio*) swimming in a Petri dish, and a mouse (*Mus musculus*) walking on a square arena and swimming in a mater maze. The raw data was either generated by the authors or kindly provided by other labs. Figure 3 illustrates in detail the application of the segmentation scheme for a fly larva, fish and mouse. The resulting movies for all organisms can be found as Movie S1.

From the contour and skeleton images, the area, the perimeter size and skeleton length are saved. This information can be used, for instance, to normalize the size of the animal, to measure rhythmic patterns of locomotion from body contractions or, potentially, to detect hunching or rearing. Coordinates including the animal's centroid position and the middle point of the skeleton are extracted as well. Next, the software translates all high-resolution coordinates (positions of head, tail, centroid, midpoint, ordered skeleton from head to tail, and contour) to the laboratory frame of reference, and it converts these features from pixels to units of physical length. Calibration implies a multiplication of the coordinates by a conversion factor calculated from landmarks of the arena available in the field of view. To define customized landmarks, the code displays the arena background on the screen and asks the user to click on key positions separated by distances known to the experimenter. The spatial scale (pixels per millimeter) and the temporal resolution (frames per second) of the tracking are saved together with the arena landmarks.

### High-resolution sensorimotor trajectories

**Figure 4:**
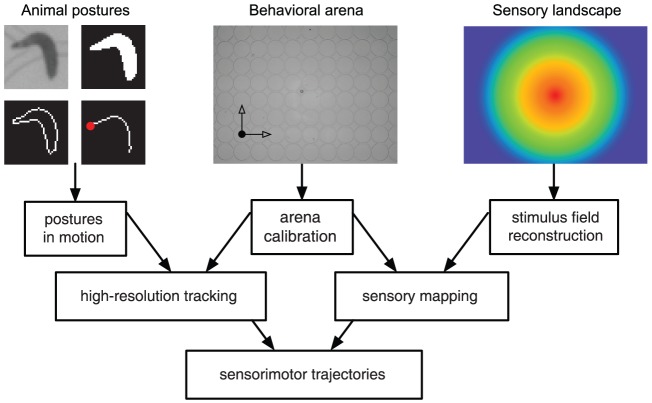
High-resolution sensorimotor trajectories. The posture and position of each animal are mapped onto the arena system of reference and then used to infer, from the gradient landscape, the sensory dynamics at different loci of interest. The temporal sequences of all sensory and motor variables correspdonding to different animals are compiled in a single data file.

In the presence of a stimulus landscape or during a particular behavioral task, postural data can be used to infer the sensory input to which the animal is exposed during the course of an experiment. This yields to detailed sensorimotor trajectories. By projecting the body shapes onto the plane of locomotion and calculating postures in time and space, we can map the position of the animal's head with the corresponding stimulus intensity and local gradient's strength and direction. Aligning the motor data with the reconstructed stimulus landscape, we can obtain, for instance, the stimulus intensity at the head, its time derivative and the relative orientation angle of the animal with respect to the local gradient (Figure 4).

After testing several animals independently (for instance corresponding to the same sensory stimulus, genotype, developmental stage, etc.), our offline analysis software allows for merging files from all trials in a consistent way. Together with continuous kinematic variables, *SOS* detects and saves discrete behavioral events such as runs, turns and casts, by finding abrupt reorientation speeds and large head bending angles [21]. On the whole, the process produces a temporal sequence of high-resolution sensory and behavioral data for all animals tested. Hypothesis about neural computation can then be drawn from statistical correlations between sensory inputs and motor outputs.

### Orientation in sensory landscapes: chemotaxis, thermotaxis and phototaxis in the *Drosophila melanogaster* larva

**Figure 5:**
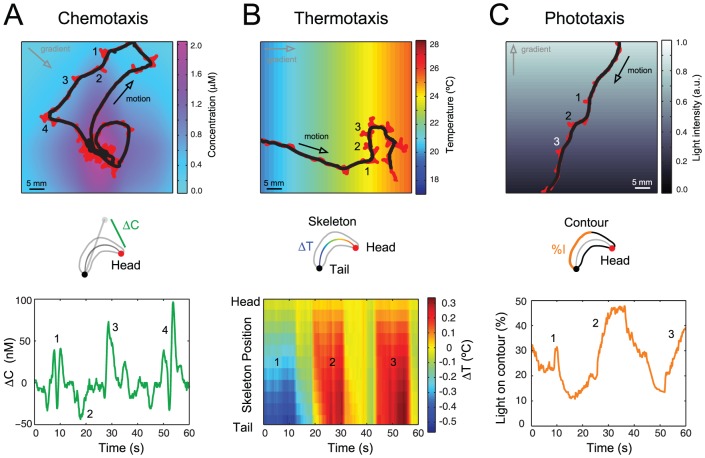
*Drosophila* larval orientation in odor, temperature and light gradients. Top: stimulus landscape overlaid with one representative head-and-tail trajectory for every sensory modality. Bottom: reconstructed time course of a sensory variable relevant to the behavioral modality under study. (**A**) Concentration changes due to head casts as the animal returns to the odor source. (**B**) Thermal differences along the body axis skeleton with respect to the head as the animal reorients in the temperature gradient. (**C**) Percentage of light on the body contour as the larva moves away from the light source. For all panels, gradient direction and direction of motion are illustrated with grey and black arrows, respectively. Numbers indicate the occurrence of sampling events reported in the bottom panel.

From online tracking to offline processing and the generation of sensorimotor trajectories, we illustrate the potential of the whole *SOS* system by examining *Drosophila* larvae orienting to odor, temperature and light gradients [22]. Our present aim is not to conduct an exhaustive study of each modality, but to show how the analysis can reveal interesting aspects of sensory orientation in the larva. The trajectory of particular points along the body is used to reconstruct both the positional dynamics of the sensory organs and the behavior of the entire animal. As shown in Figure 5, we infer the sensory input at the body locations where receptors are located.

*Drosophila* larvae move towards increasing concentrations of attractive odors [12], [17], [21], [23], [24], [25]. The larval “nose” is located at the tip of the head, where a pair of olfactory organs (dorsal organs) host 21 olfactory sensory neurons [15], [26]. It has been shown that larval chemotaxis does not require stereo-olfaction, namely, the detection of concentration differences between the left and the right “noses” [17]. Reconstruction and analysis of the odor concentration dynamics at the tip of the head during free exploration has shown that head casts are a key process of the reorientation mechanism [21]. Such trajectories revealed that temporal integration of stimulus changes during head motion represents the main input signal controlling the timing and direction of turns. Lateral head sweeps constitute an active sampling process that allows the animal to reorient in the odor gradient [21]. As quantified in Figure 5A, lateral head movements can be associated with rapid changes in odor concentration on the order of a 5% relative difference (50 nM changes in a 1 µM concentration background).

*Drosophila* larvae sense temperature by using a pair of detectors located at the tip of the head (terminal organs) and chordotonal neurons scattered along the body wall [14]. When exposed to a temperature gradient, first instar larvae modulate the acceptance of a new direction of motion during lateral head sweeps [27]. As argued in reference [14], thermal equilibration from the surface to the internal structure of first instar larvae should take place in less than a tenth of a second. Applying the same scaling argument to third instar larvae, we find that a few seconds are necessary for thermal equilibration along the lateral axis of the body. In contrast, the characteristic time for thermal equilibration along the longitudinal axis (4 mm) is nearly one minute. This slow timescale implies that, in principle, third instar larvae could measure temperature differences along the antero-posterior axis. By reconstructing the sensorimotor trajectory of a larva navigating from low to high temperatures in a linear temperature gradient of slope 0.1°C/mm, *SOS* provides a quantitative estimate of the temperature differences along the larva's body during orientation. As shown in the Figure 5B, we find that variations along the anteroposterior axis are of the same order of magnitude than those associated with head casts.

*Drosophila* larvae display a strong photophobic behavior lasting until wandering stage [28]. Two sensory organs (Bolwig's organs) are located in the head. Moreover, non-conventional photoreceptors tile the entire body wall [16]. From genetic dissections of light driven behaviors in the fruit fly larva [29], [30], [31], [32], [33], there is a growing interest in the quantification of orientation responses in light gradients [34]. Instead of exposing individuals to all-or-none light flashes, we tested larval behavior in response to a sideways light gradient (Figure 5C). The orientation mechanism controlling light responses is thought to involve body bends or head casts [28]. Turning direction might also be inferred from a comparison of the light exposure on the left and on the right sides of the body. In our arena the animal controls light exposure by modifying its orientation with respect to the direction of the source. In Figure 5C, we use *SOS* to quantify the temporal evolution of the percentage of body surface exposed to light. Together with the light intensity at the Bolwig's organs, our system allows us to estimate subtle differences in illumination along the left and right sides of the body — a possible parallel source of information exploited by the larva during phototaxis.

## Discussion

Adaptive behavior refers to the ability of an animal to produce and react to changes in internal and external signals by means of motion [35]. At the same time, behavior often implies an active process to collect sensory information, rather than a passive response. Motion, perception, and proprioception are not independent but are interwoven in a sensorimotor feedback loop. With the aim of improving and sharing behavioral quantification tools, we have developed software for high-resolution tracking of single animals that are freely moving in two-dimensional sensory environments. By monitoring the postural changes of an individual over time, our system reveals the stimulus history to which specific sensors are exposed in space and time. This reconstruction of trajectories in sensory and motor spaces represents a necessary step in the analysis of the neural processes controlling active sampling during orientation behavior.

Across the animal kingdom, quantitative measures of motor responses have provided invaluable information about the computation underlying orientation behavior [12], [36], [37], [38], [39]. Historically, the advent of high-resolution tracking of *Escherichia Coli* in chemical gradients [40], [41] laid the foundation for an understanding of the biochemical pathways controlling chemotaxis in unicellular organisms. Similar behavioral analysis conducted in *Caenorhabditis elegans* [42], [43], [44] and in *Drosophila melanogaster* larvae [17], [21], [27] have shed light on the neural mechanisms controlling active sampling and decision making. Here, we demonstrate the use of our tracking software on three sensory modalities in the fruit fly larva: chemotaxis, thermotaxis and phototaxis. Our analysis produces behavioral features associated with the detection and computation of sensory stimuli in each modality: odor concentration changes due to side-to-side head movements during chemotaxis; temperature gradients along the larval body during thermotaxis; and differences in photostimulation between the left and right sides of the animal during phototaxis.

The software *SOS* is a tracking and analysis system that can be used in behavioral screens to characterize subtle sensorimotor deficiencies associated with selected phenotypic traits. Precise behavioral tracking is also convenient for phenotypic scoring in genetic mapping studies [45]. *SOS* can be adapted to make use of anatomical features specific to the organism under study such as sharp edges, large protrusions, darker parts, and expression of fluorescent markers or tags. It can also be extended to analyze orientation behavior in other sensory landscapes such as humidity, gravity, or simply foraging strategies in homogeneous sensory landscapes. Finally, *SOS* can be applied to monitor a wide range of organisms from *Planarian* [46] and marine zooplankton [47] to larvae from other species [48], fishes and mice. We hope that novel ways of measuring and analyzing animal behavior will contribute to the development of new concepts, theories and principles about how neurons process sensory information to produce coordinated motor responses [49].

## Supporting Information

Text S1 **Step-by-step tutorial detailing the use and functionality of** ***SOS*** **.**
(PDF)

Movie S1 **Illustrative movie of posture tracking in flatworms, fruit fly larvae, fishes and mice.**
(M4V)

File S1 **Tracking and analysis codes of** ***SOS*** **together with a test dataset generated from larvae behaving in an odor gradient.** Updated code versions will be uploaded on the website of the Louis lab: http://www.crg.es/matthieu_louis.
(ZIP)
