# Correction: Automated Tracking of Animal Posture and Movement during Exploration and Sensory Orientation Behaviors

**DOI:** 10.1371/annotation/5bef5b0a-9b48-4e85-8df5-a46793f6c701

**Published:** 2012-10-04

**Authors:** Alex Gomez-Marin, Nicolas Partoune, Greg J. Stephens, Matthieu Louis

Figure 2 had numerous errors. The correct figure can be found here: 

**Figure:**
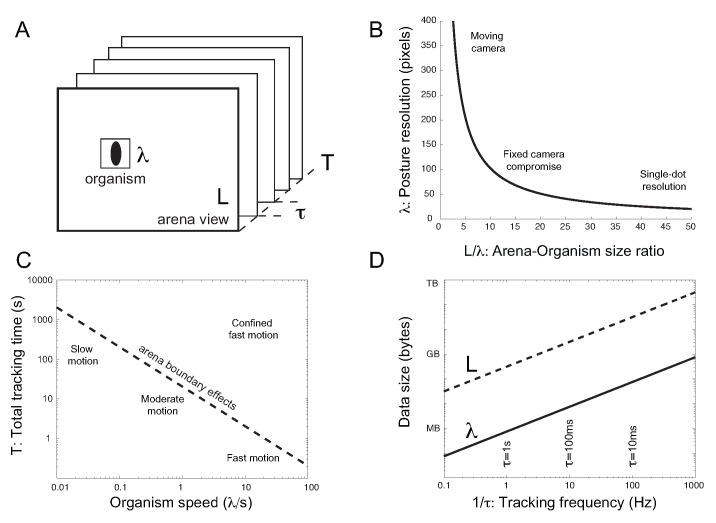


.

