# Supplementary material for: Automated Tracking of Animal Posture and Movement during Exploration and Sensory Orientation Behaviors
Source: PLoS One. 2012 Aug 9;7(8):e41642. doi: 10.1371/journal.pone.0041642 (PMC3415430; doi:10.1371/journal.pone.0041642)
Supplement: Text S1 — Step-by-step tutorial detailing the use and functionality of SOS . (PDF) [file pone.0041642.s001.pdf]

SOS

Sensory Orientation Software

Supplementary Guide

Index

- License
- Requirements
- Material
- Input/output files
- Step-by-step instructions
- Example test data set
- Supplementary movie

License

Under a Creative Commons Attribution License, SOS is a collection of scripts developed for animal tracking and behavioral analysis. It is made public in the hope that it will be useful without any warranty. You are welcome to redistribute and modify it according to your needs. If you publish or present results that are based on any of the scripts or ideas, fully or partly, please acknowledge and cite the original open-access publication:

Automated tracking of animal posture and movement during exploration and sensory orientation behaviors. Gomez-Marin et al. PLoS ONE *2012.*

Requirements

SOS software are Matlab-based scripts, organized in series of online and offline routines. The routines were developed and tested with the version of Matlab R2009a. SOS online tracking requires the Image Acquisition and Image Processing toolboxes. Furthermore, it requires a FireWire camera and its connection cable, a behavioral arena with proper illumination and, importantly, an animal to track. SOS offline only needs the Image Processing toolbox. It can be run on experimental data collected and preprocessed by SOS online or, alternatively, it can use already existing movie files offline from previous experiments.

Material

As explained, described and detailed in the main publication, the SOS software is organized in two main modules:

1. **SOS online** for behavioral tracking and image preprocessing.
2. **SOS offline** for high-resolution sensory-motor processing and analysis.

The first module, SOS online, converts frames streamed live from the camera into a sequence of small files capturing the animal posture moving in space and time. It is run by the master function:

(1.1) **track.m**

The second module is run offline and consists of four main scripts:

(2.1) **loci.m** finds loci from the animal's posture and skeleton (from SOS online data).
(2.1b) **locib.m** finds head and tail from body curvature (from any existing video frames).
(2.2) **merge.m** merges the data for all experiments.
(2.3) **motion.m** calculates relevant kinematic parameters as motor output data.
(2.4) **sensation.m** maps motor points to the landscape to infer sensory experience.

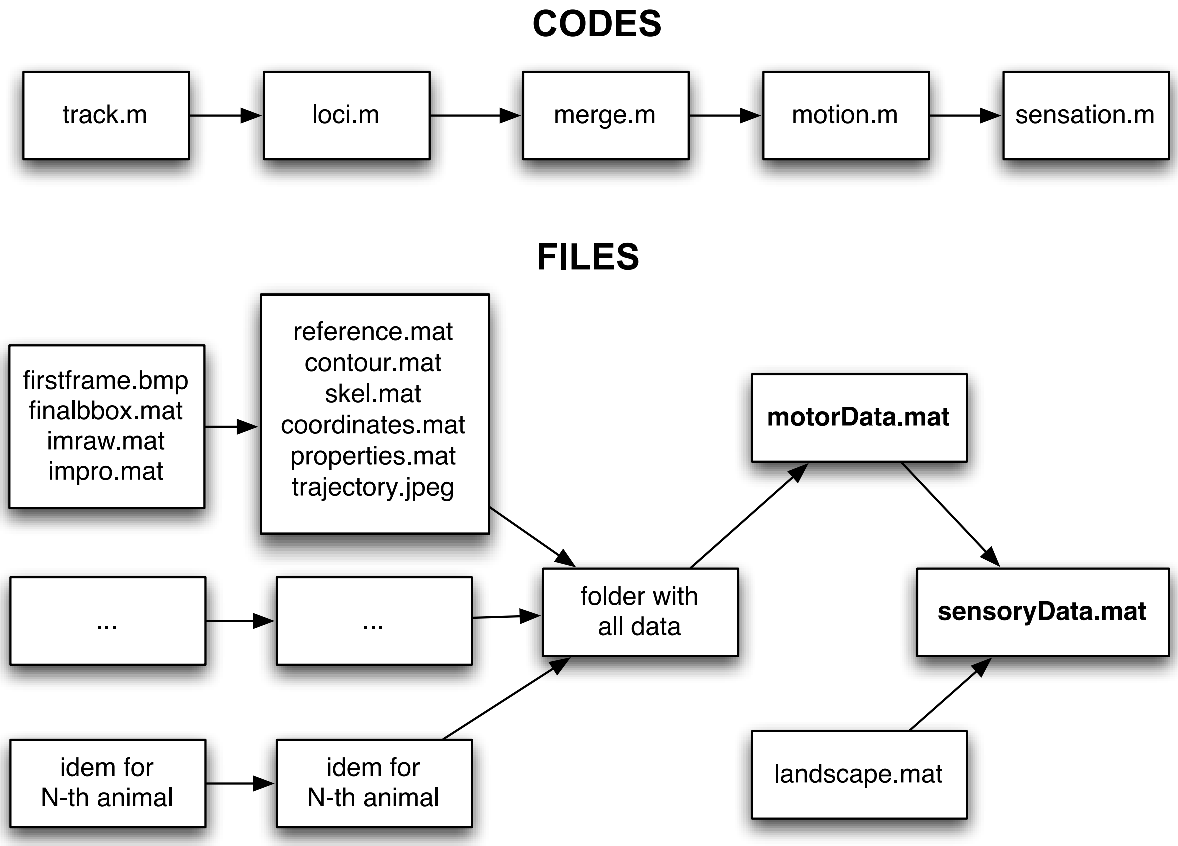

Figure 1. SOS flow scheme and sequential use of online and offline script codes, together with their corresponding input and output files.

Input/output files

track.m generates the following output files upon processing of the images streamlined from the camera:

- ***firstframe.bmp****:* initial whole field-of-view image of the animal in the arena.
- ***finalbbox.mat***: bounding box coordinates in time for laboratory reference frame.
- ***imraw.mat****:* time series of cropped raw images around the animal.
- ***impro.mat***: time series of cropped black-and-white images around the animal.

loci.m reads the output data from **track.m** and generates:

- ***contour.mat****:* time series of the animal contour positions.
- ***skel.mat***: skeleton of the animal as a function of time.
- **coordinates.mat**: time series of positions of loci of interest such as head, tail or centroid.
- **trajectory.jpeg**: snapshot of the whole high-resolution postural trajectory of the animal.
- ***properties.mat***: scalar values such as the animal's area, perimeter or skeleton length.
- ***reference.mat***: arena landmarks and spatial and temporal scales.

locib.m reads a sequence of frames from any existing video data (which, therefore, does not need to have been generated with SOS online) and extracts the postural descriptors of the animal. Head and tail loci are found from the animal's curvature, as an alternative or complementary method to the skeletonization procedure implemented in **loci.m**.

merge.m copies and pastes the above data files in a common folder, numbering each trial as single-animal experiment. From then on, every sensorimotor variable is simultaneously processed for all times and for all animals.

motion.m runs over all files in the folder generated by **merge.m**. It outputs a unique file:

- **motorData.mat:** kinematic variables (positions, speeds and angles) and behavioral modes (run, turn, cast) for every time point and every animal behaviorally tested. The exhaustive list of the information saved in this file is available in the screenshots of Figure 15.

sensation.m reads the output data from ***motion.m*** and uses the experimental reconstruction of the sensory landscape to generate:

- **sensoryData.mat**: sensory variables (bearing angle, stimulus at the animal's head, etc). The exhaustive list of the information saved in this file is available in the screenshots of Figure 16.

Step-by-step instructions

Before running the software, we recommend to first read the original manuscript and this tutorial. All codes are commented so that one can follow what operation takes place at every line of the script. Now we follow step-by-step and visually illustrate the user-software interactions during the course of an experiment.

(A) **Getting started**

Once the camera is connected to the computer, it is useful to type *imaqhwinfo* in the command window to retrieve information about whether it is properly recognized. One should then check whether the *dcam* driver for firewire cameras is in place so that video object can be assigned to the camera.

(B) **Running track.m**

Next, set the correct file path and run the routine **track.m**, specifying the time lapse between frames and number of frames to be acquired. Automatically, a live image of the behavioral arena should be displayed on screen. Make sure the lens cap is removed and the camera diaphragm is not blocking too much or to little light. As illustrated by the next screenshot, follow the prompt instructions and introduce the animal in the arena.

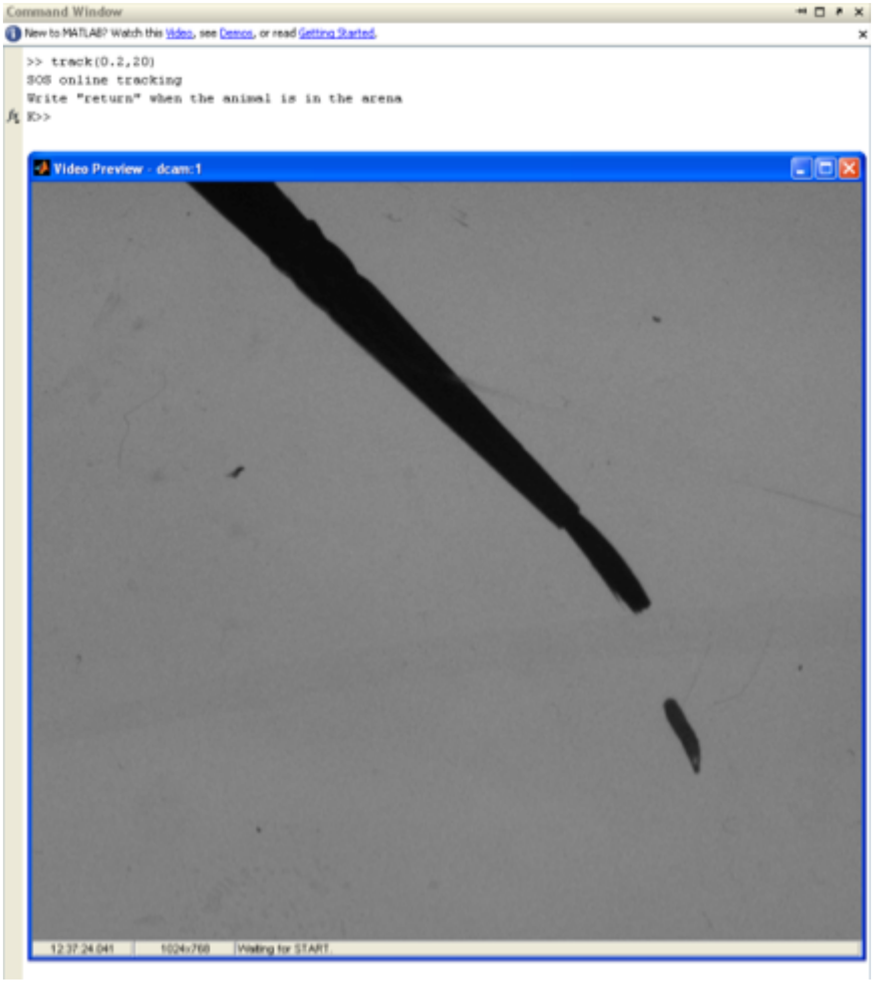

Figure 2.

As depicted in Figure 1 of the main text, different illumination conditions and threshold values crucially influence animal detection and background reconstruction. Track will call internal subroutines allowing the user to iteratively set up values before tracking starts. As explained in the main text and Figure 2, it is important to appropriately choose the field of view with respect to the animal resolution and the temporal frequency of the tracking.

Before the tracking starts, SOS online displays the image processing for animal detection corresponding to the default settings and allows the user to optimize the threshold.

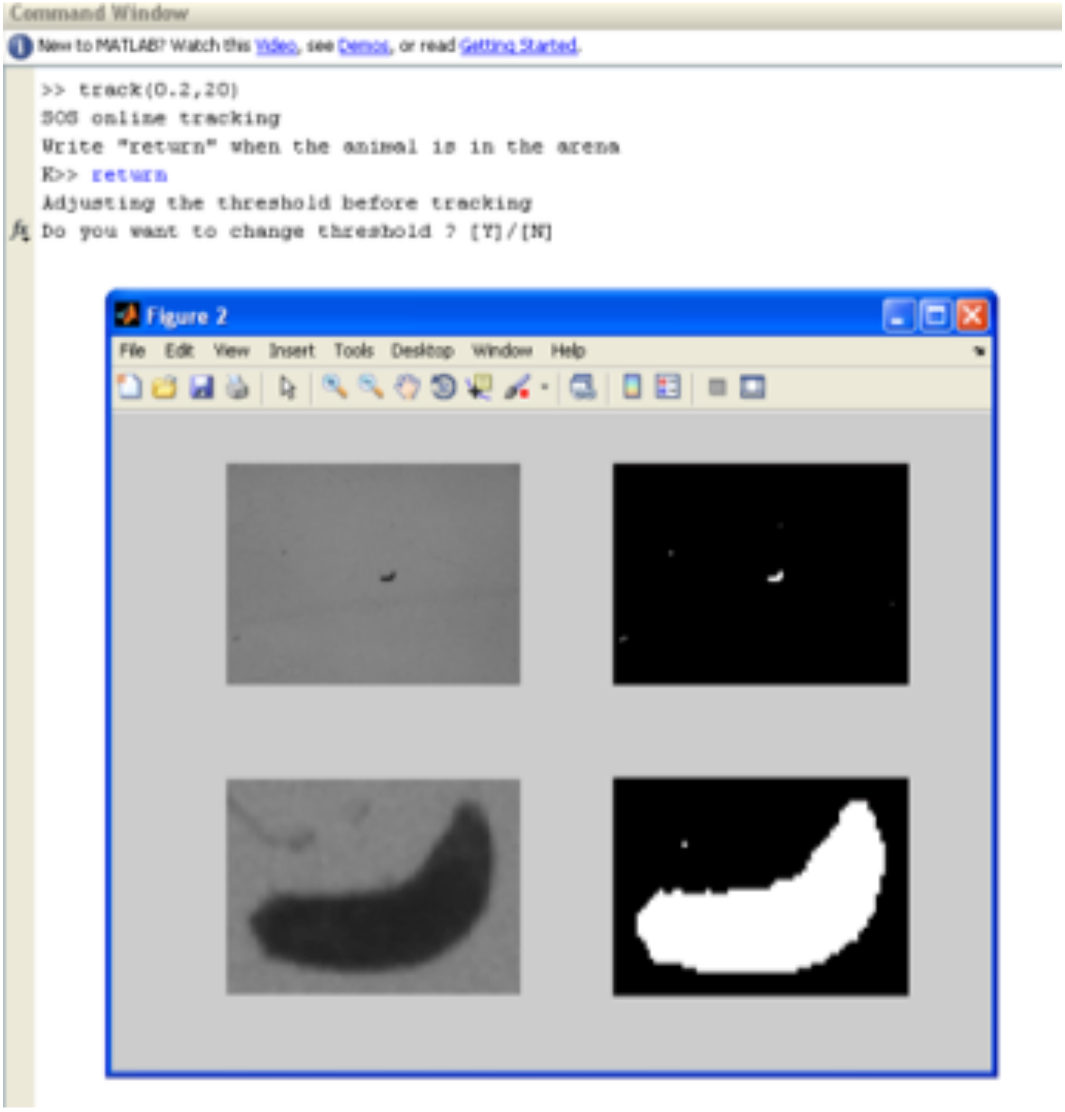

Figure 3.

As the values are changed, the software trials to detect the animal are shown on screen.

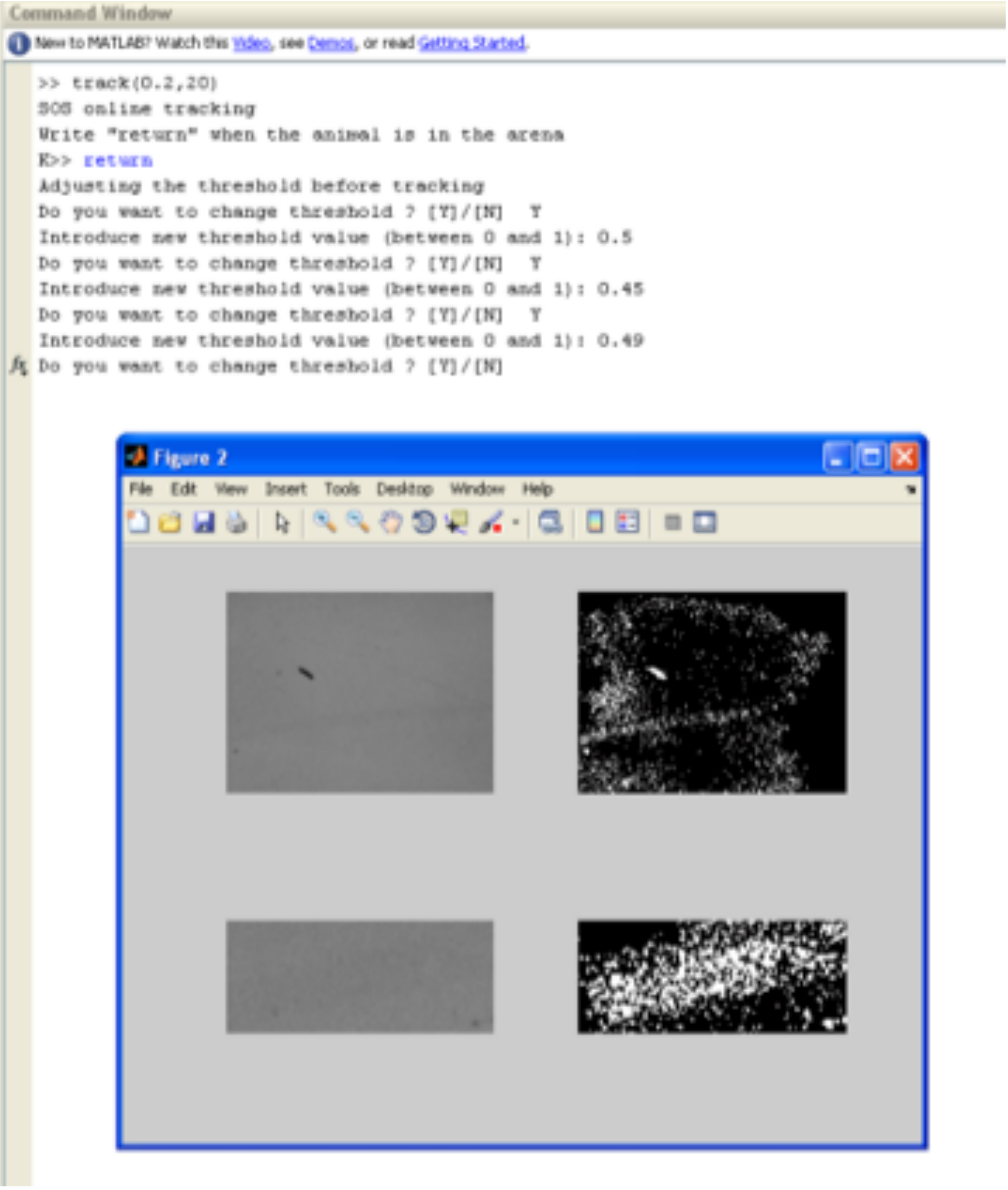

Figure 4.

When the user is satisfied with the current settings, typing “N” fixes and saves the threshold values and SOS online moves on to the following operation.

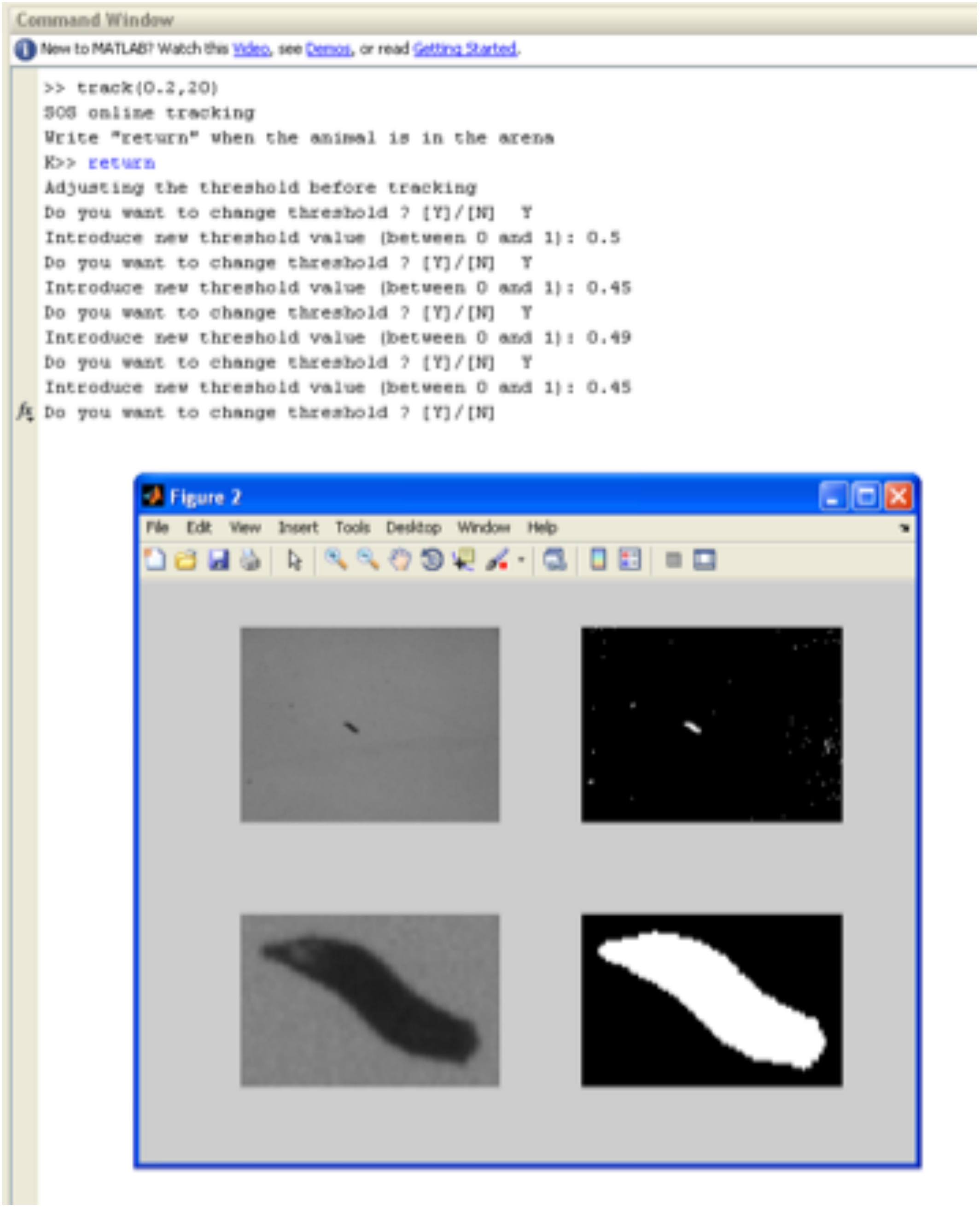

Figure 5.

Once this is done, SOS automatically reconstructs the background and the software is ready to start tracking. All these steps and the status of the tracker can easily be followed by the instructions displayed at the command window.

(C) **Running loci.m**

To run loci for offline analysis, please make sure the folder directory and path are correct

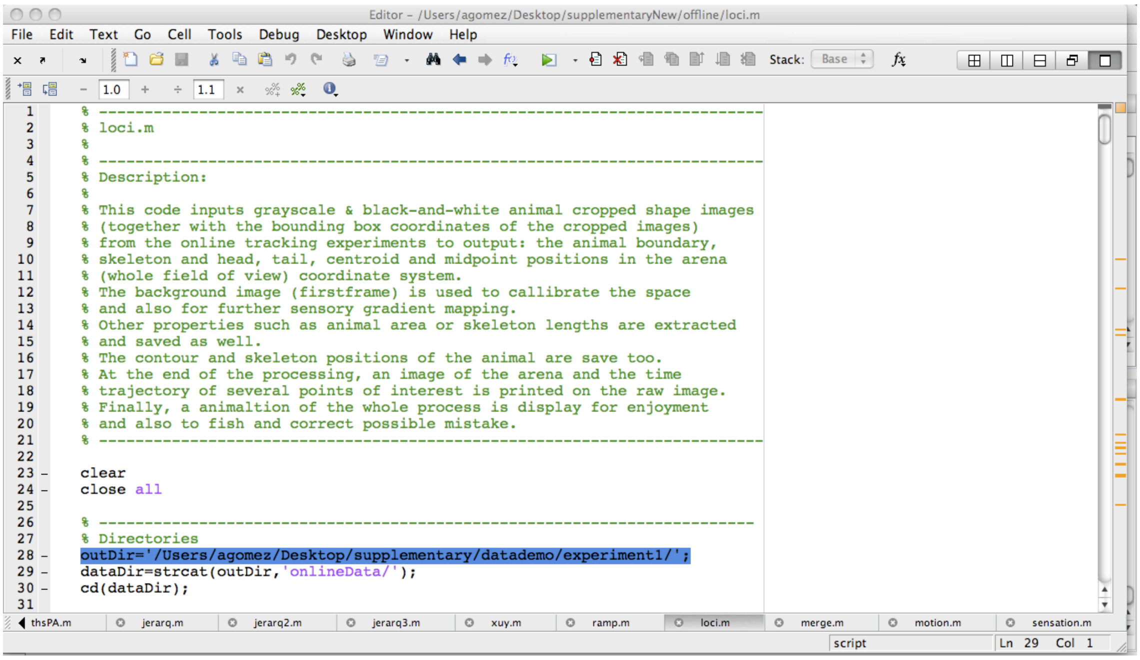

Figure 6.

First, an image is displayed asking the user to click on specific landmark points.

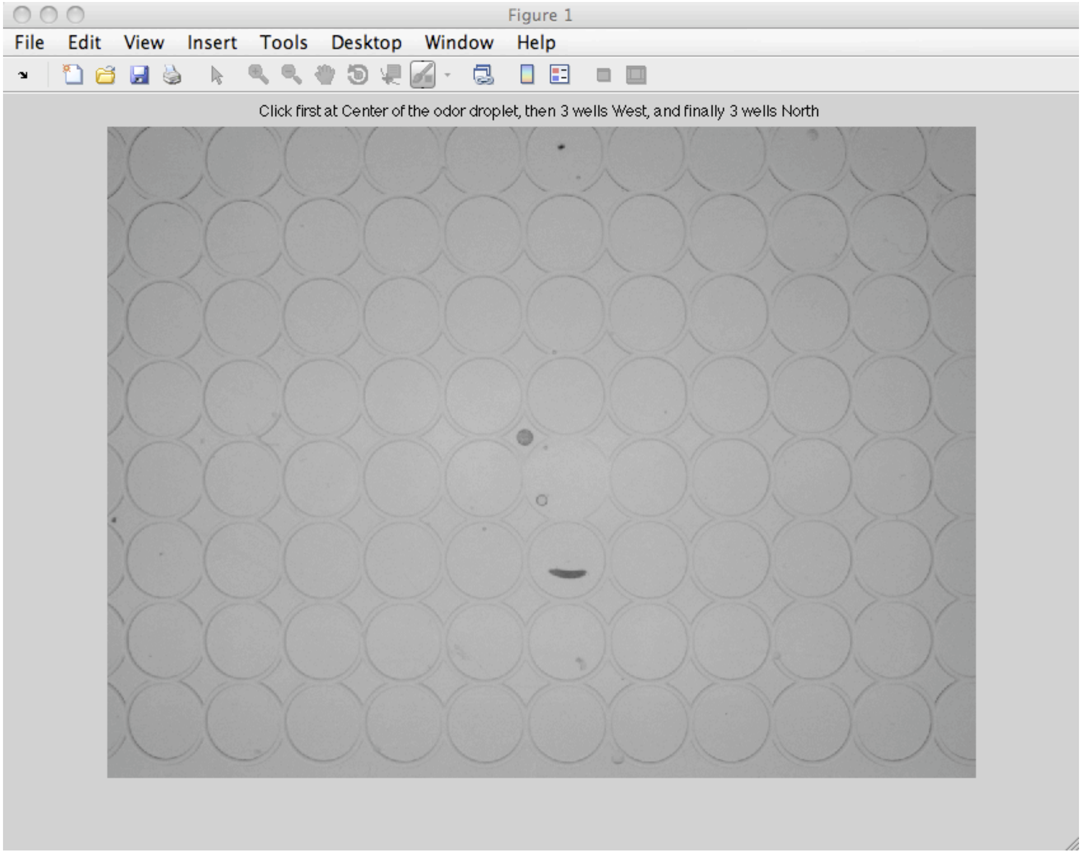

Figure 7.

After having selected 3 landmark, the user will find their positions depicted in colors for the user to validate them.

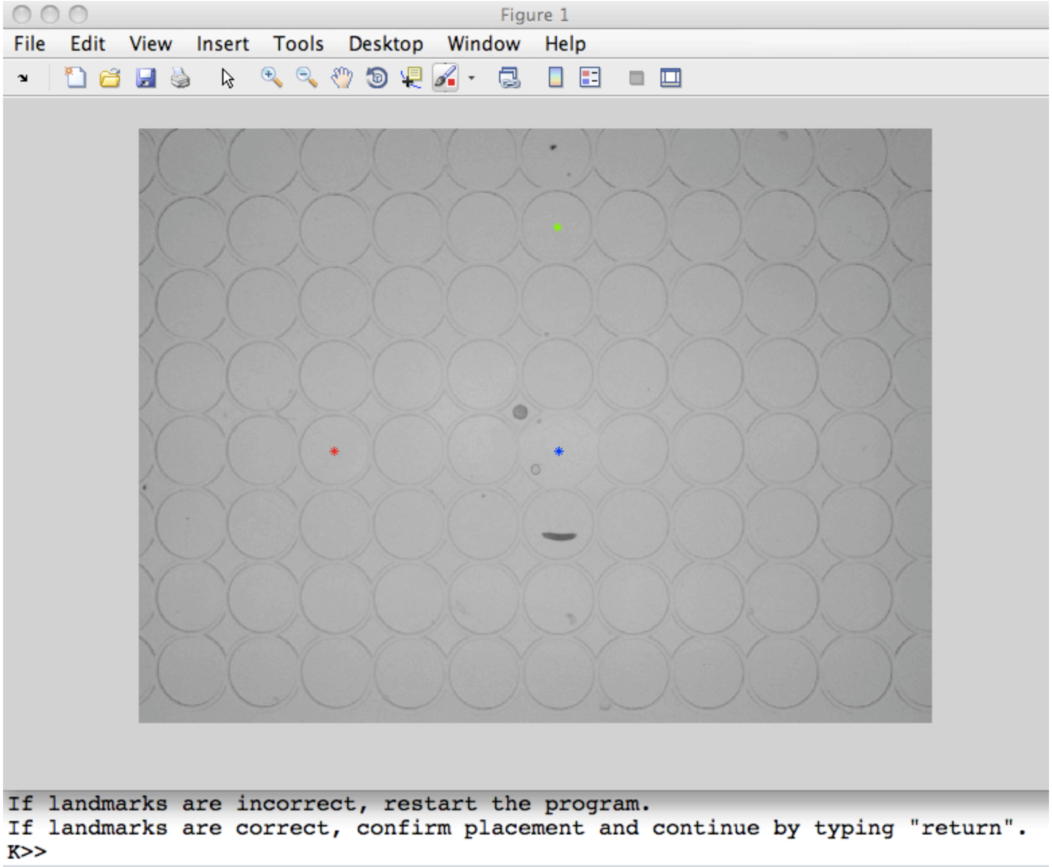

Figure 8.

Next, the first frame is shown for the user to click on animal's head and tail respectively.

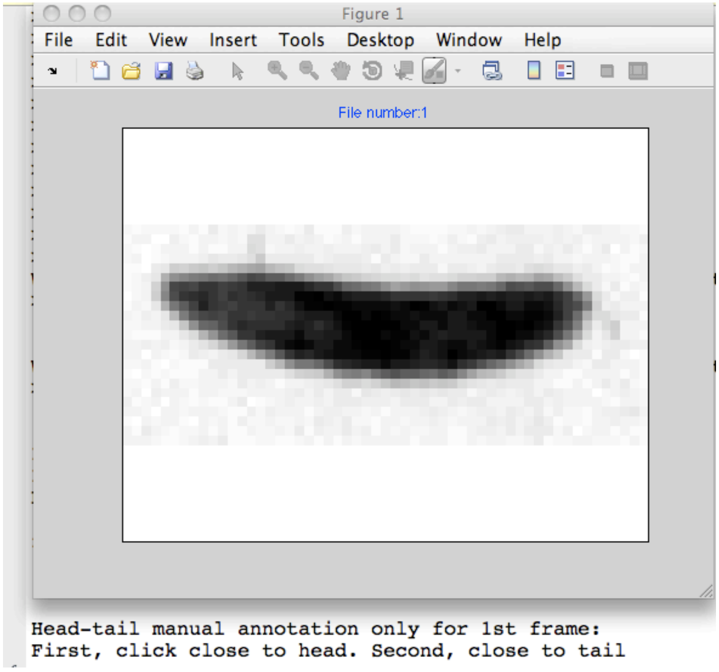

Figure 9.

After landmark and head-tail clicking, the code runs automatically through all frames, displaying the fraction of total frames processed.

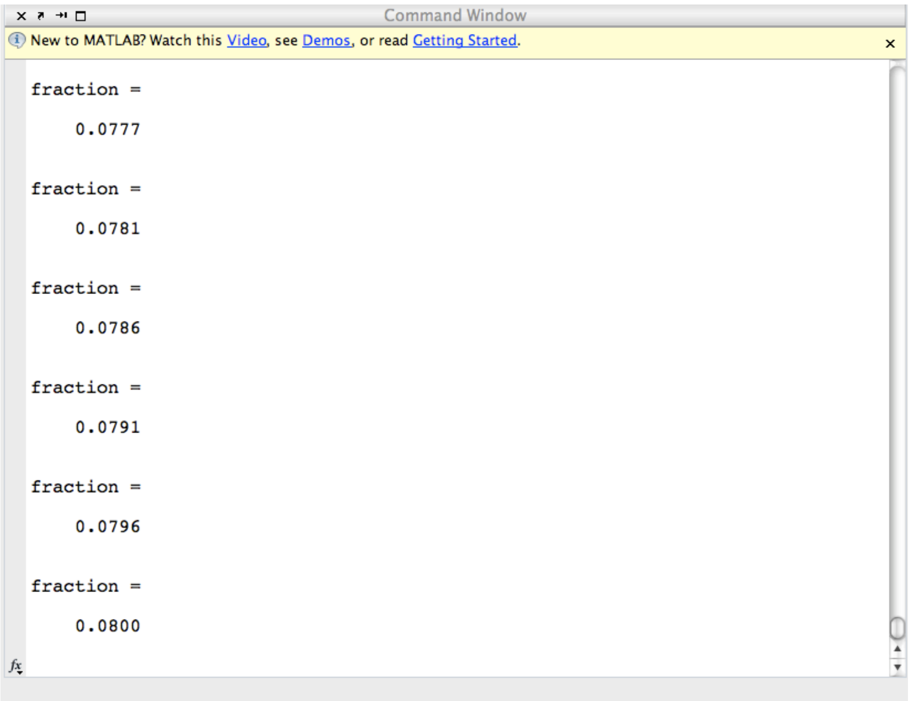

Figure 10.

When all frames have been processed, an animation of the whole sequence is displayed.

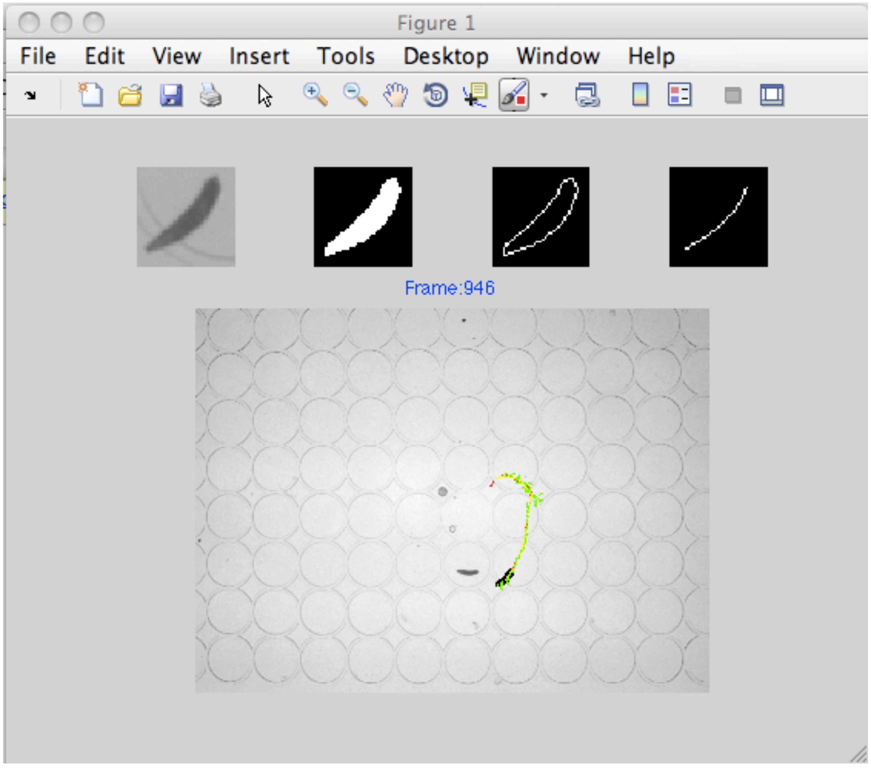

Figure 11.

If the animation reveals swaps in the head and tail trajectory, one can run the code again activating the flag to correct for head-tail swaps by reviewing potential problems such as more than two skeleton endpoints (spurs) and small animal aspect ration (blobs). At these frames, head-tail manual annotation is required.

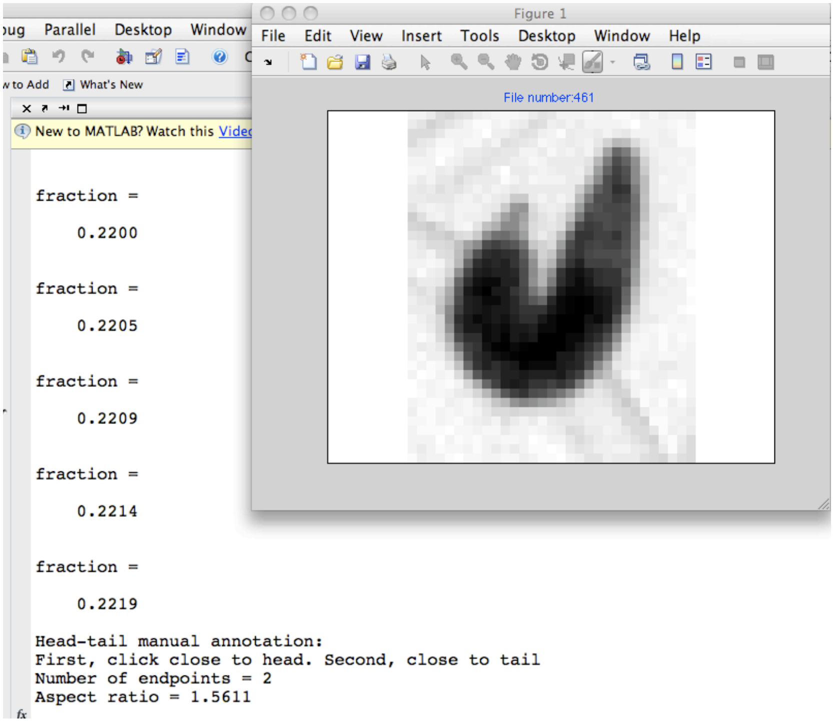

Figure 12.

In the end**,** head, tail, midpoint and centroid trajectories are shown and saved.

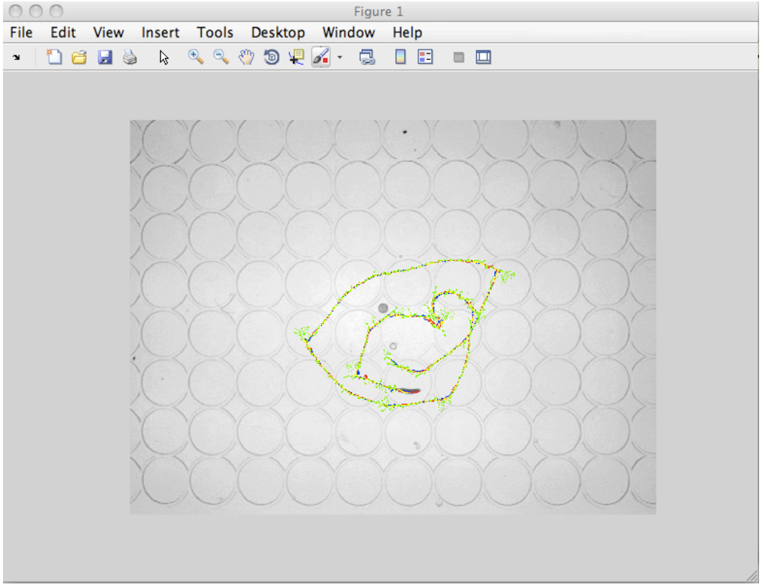

Figure 13.

(D) **Running merge.m**

All files generated by **loci.m** are copied and pasted together for every animal trial.

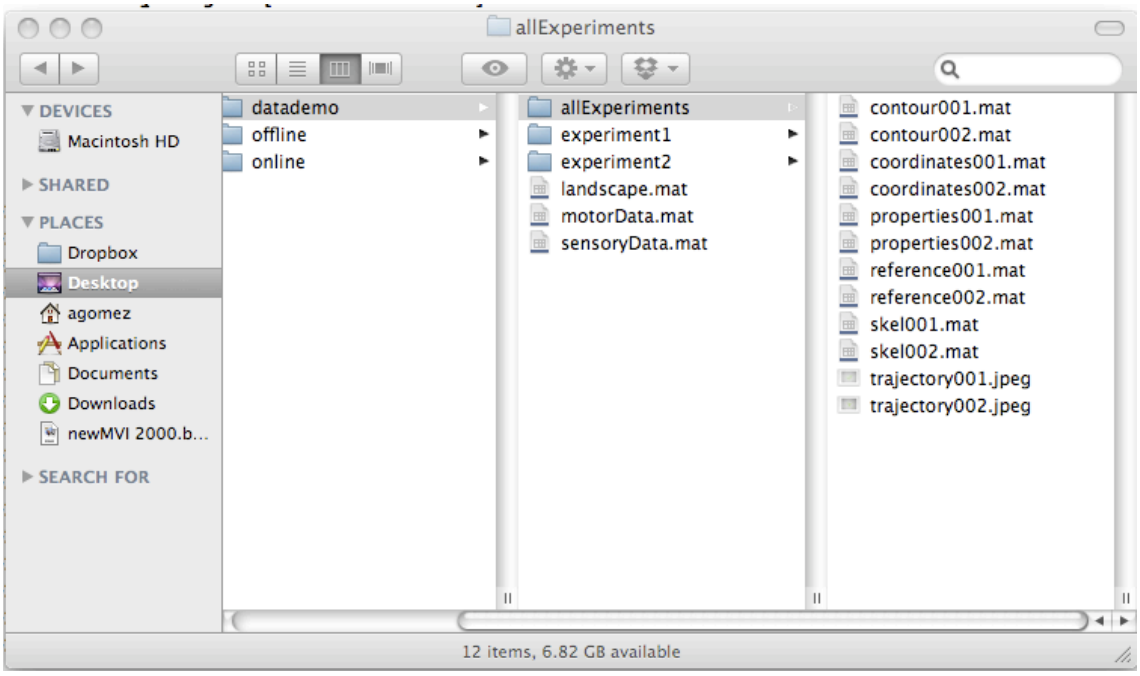

Figure 14.

(E) **Running motion.m**

For all times and all animals, motor data is generated and compiled in a single file array.

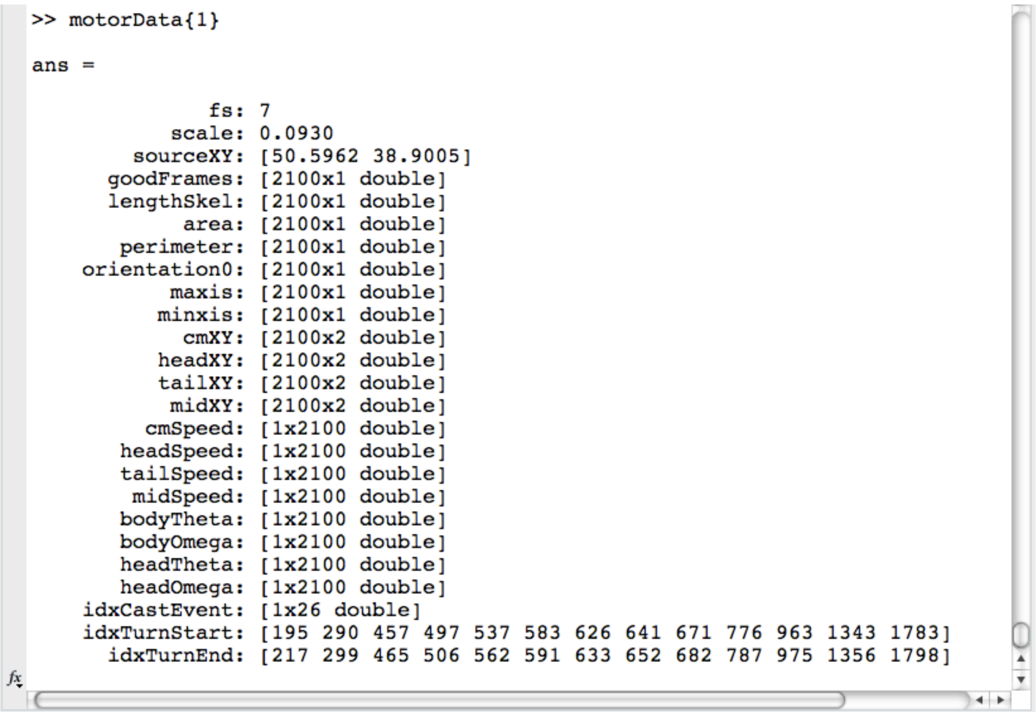

```
>> motorData{1}

ans =

             fs: 7
          scale: 0.0930
       sourceXY: [50.5962 38.9005]
     goodFrames: [2100x1 double]
     lengthSkel: [2100x1 double]
           area: [2100x1 double]
      perimeter: [2100x1 double]
   orientation0: [2100x1 double]
          maxis: [2100x1 double]
         minxis: [2100x1 double]
           cmXY: [2100x2 double]
         headXY: [2100x2 double]
         tailXY: [2100x2 double]
          midXY: [2100x2 double]
        cmSpeed: [1x2100 double]
      headSpeed: [1x2100 double]
      tailSpeed: [1x2100 double]
       midSpeed: [1x2100 double]
      bodyTheta: [1x2100 double]
      bodyOmega: [1x2100 double]
      headTheta: [1x2100 double]
      headOmega: [1x2100 double]
   idxCastEvent: [1x26 double]
   idxTurnStart: [195 290 457 497 537 583 626 641 671 776 963 1343 1783]
     idxTurnEnd: [217 299 465 506 562 591 633 652 682 787 975 1356 1798]
```

Figure 15.

(F) **Running sensation.m**

Similarly, different sensory variables are generated and compiled in another file array.

```
>> ls
allExperiments  experiment2    motorData.mat
experiment1     landscape.mat  sensoryData.mat

>> load sensoryData.mat
>> sensoryData{1}

ans =

           headSens: [1x2100 double]
             cmSens: [1x2100 double]
           tailSens: [1x2100 double]
            midSens: [1x2100 double]
        headSensDot: [1x2100 double]
          cmSensDot: [1x2100 double]
        tailSensDot: [1x2100 double]
         midSensDot: [1x2100 double]
    headSensDotNorm: [1x2100 double]
      cmSensDotNorm: [1x2100 double]
    tailSensDotNorm: [1x2100 double]
     midSensDotNorm: [1x2100 double]
           headGrad: [1x2100 double]
             cmGrad: [1x2100 double]
           tailGrad: [1x2100 double]
            midGrad: [1x2092 double]
            stimDir: [1x2100 double]
            bearing: [1x2100 double]

fx >>
```

Figure 16.

Test dataset

As supplementary material we provide a test data set from real experiments for the user to practice and get familiar with every offline subroutine. Let's assume you performed experiments on larval chemotaxis behavior and the tracking data for every single-animal experiment is in its corresponding folder. SOS can automatically generate high-resolution sensorimotor trajectories. Following the instructions below, one can recreate the data analysis process.

1. For each experiment, run **loci.m** on the files in the onlineData folder. These were generated during the data collection by SOS online track. Remember to set the appropriate Matlab paths and directories. We provide a second experimental animal trial. Run routine **loci.m** on both folders. See previous instructions and snapshots for details. Pay attention to the end of the processing, where the head-tail reconstruction is animated.

2. Then run **merge.m** in order to merge these two animal tracks in a common folder with their corresponding files numbered by individual.

3. Third, run **motion.m** on that data to calculate and save the most relevant kinematic variables and behavioral states for every time point and every animal.

4. Finally, run **sensation.m** whose input is the motor data and the experimental reconstruction of the sensory landscape to which the animal was exposed (also provided).

The exercise ends once you have generated motor and sensory trajectories from the experimental cropped animal postures.

Supplementary movie

As an additional illustration of SOS software applicability to different behaviors and organisms, we include a supplementary movie of high-resolution posture tracking of a swimming flatworm, crawling larva, swimming fish, walking mouse and swimming mouse in their corresponding behavioral arenas during free orientation or exploration.
