# Supplementary figures and images for: Automated Tracking of Animal Posture and Movement during Exploration and Sensory Orientation Behaviors
Source: PLoS One. 2012 Aug 9;7(8):e41642. doi: 10.1371/journal.pone.0041642 (PMC3415430; doi:10.1371/journal.pone.0041642)

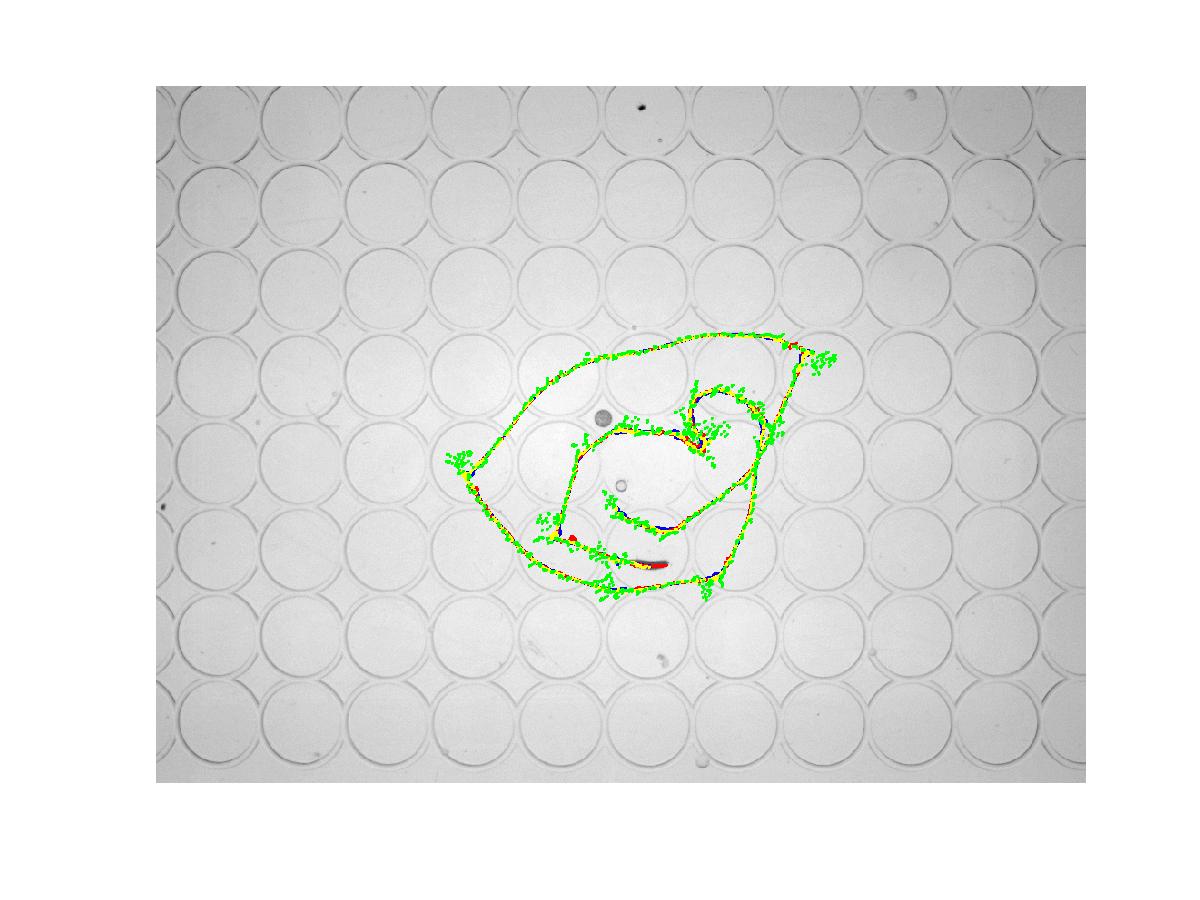

Supplement: File S1 — Tracking and analysis codes of SOS together with a test dataset generated from larvae behaving in an odor gradient. Updated code versions will be uploaded on the website of the Louis lab: http://www.crg.es/matthieu_louis. (ZIP) [file pone.0041642.s003.zip › FINAL_SOSCodes/datademo/allExperiments/trajectory001.jpeg]

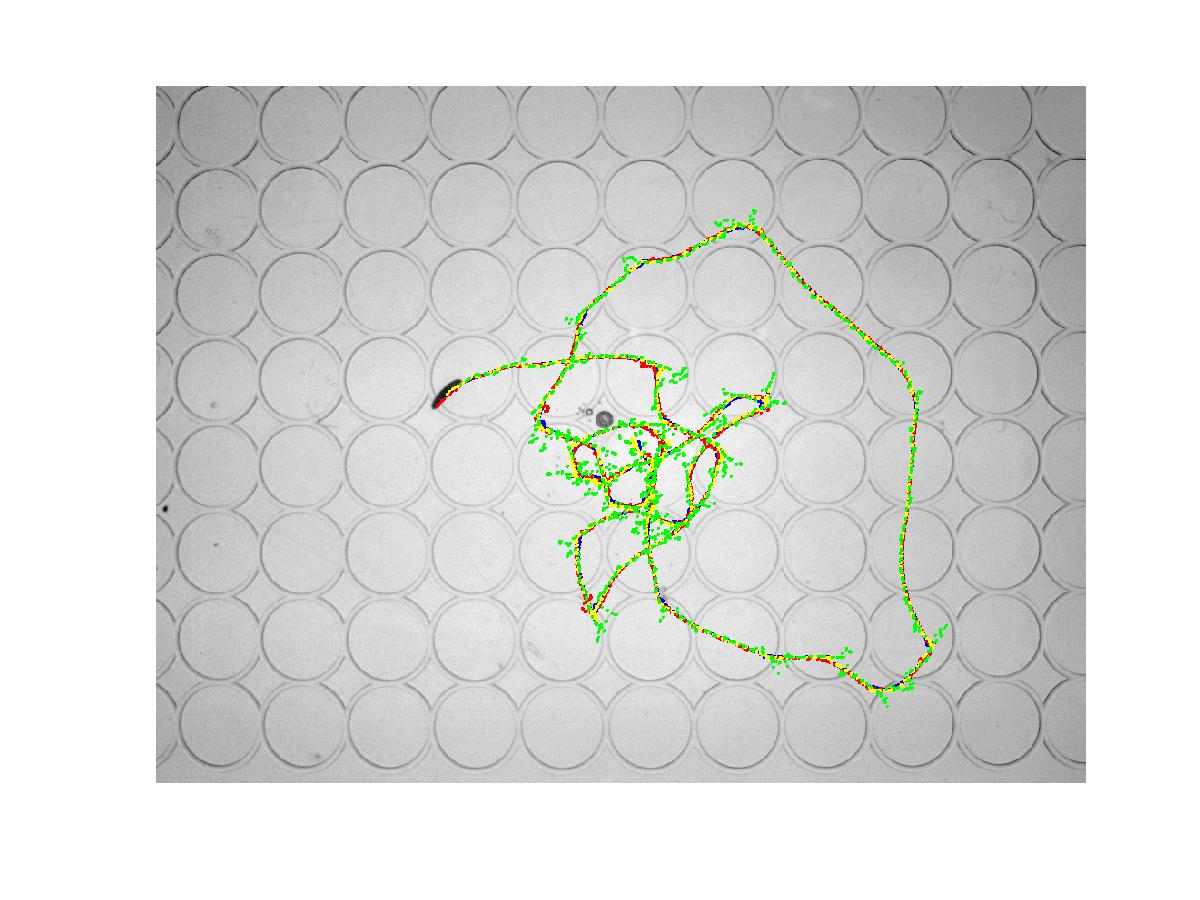

Supplement: File S1 — Tracking and analysis codes of SOS together with a test dataset generated from larvae behaving in an odor gradient. Updated code versions will be uploaded on the website of the Louis lab: http://www.crg.es/matthieu_louis. (ZIP) [file pone.0041642.s003.zip › FINAL_SOSCodes/datademo/allExperiments/trajectory002.jpeg]

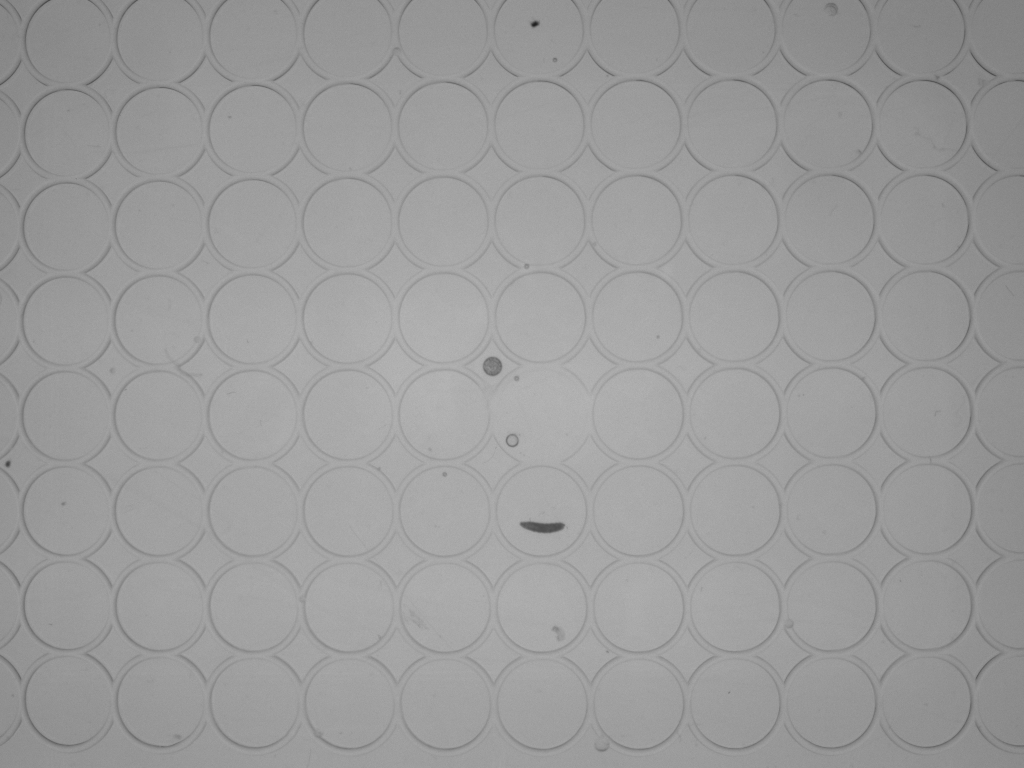

Supplement: File S1 — Tracking and analysis codes of SOS together with a test dataset generated from larvae behaving in an odor gradient. Updated code versions will be uploaded on the website of the Louis lab: http://www.crg.es/matthieu_louis. (ZIP) [file pone.0041642.s003.zip › FINAL_SOSCodes/datademo/experiment1/onlineData/firstframe.BMP]

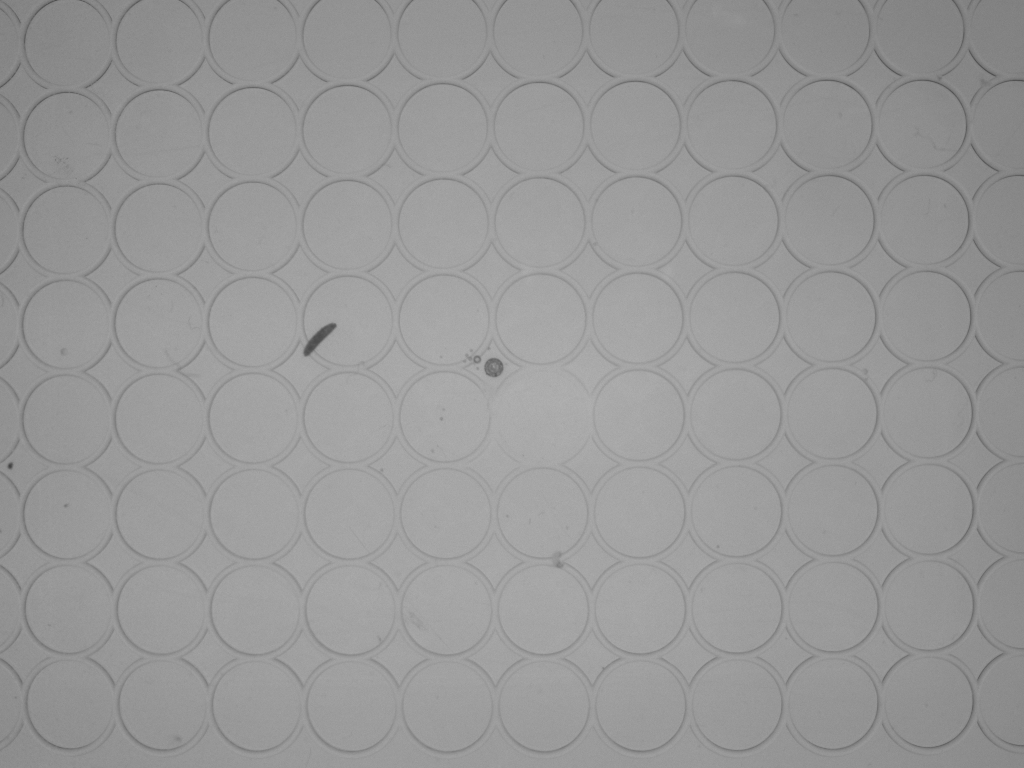

Supplement: File S1 — Tracking and analysis codes of SOS together with a test dataset generated from larvae behaving in an odor gradient. Updated code versions will be uploaded on the website of the Louis lab: http://www.crg.es/matthieu_louis. (ZIP) [file pone.0041642.s003.zip › FINAL_SOSCodes/datademo/experiment2/onlineData/firstframe.BMP]
